# Summer Clothing

**Published:** 1870-08

**Authors:** 


### SUMMER CLOTHING.

FOR all persons, especially invalids, and those who take cold easily, a thin material of woolen gauze, next the skin, is safest and best, because —

First, it is a non-conductor, carries heat from the body more slowly than cotton, linen, or silk; all colds are caused by the body becoming colder than natural, especially if it is made colder rapidly, and woolen material next the skin is the best thing known to prevent this rapid cooling, especially after exercise which has caused perspiration, and does not cause that disagreeable sepulchral dampness which wet linen does when it comes in contact with the skin.

The warmer the weather, the more need for woolen next the skin; hence British sailors are required to wear woolen next the skin, in tropical latitudes, in summer, as the best observed protection against disease.

All garments worn next the skin during the day should be removed at night and spread out for thorough airing and drying.

Cotton is the best material to be worn next the skin at night. All changes from a heavier to a lighter clothing in summer, should be made by putting on the lighter clothing at the first dressing in the morning.

It is greatly safer for children, for invalids and old persons, to have too much clothing, than too little.

